# Sedation Strategies, Drugs, and Protocols Used in Catheter Ablation of Atrial Fibrillation: A Focused Review

**DOI:** 10.31083/RCM49029

**Published:** 2026-05-26

**Authors:** Bruna Catuzzo, Ilaria Meynet, Silvia Denti, Claudia Amellone, Giuseppe Coppola, Silvana De Bonis, Martina Nesti, Pier Luigi Pellegrino, Antonio Rossillo, Matteo Ziacchi, Giuseppe Boriani, Antonio D’Onofrio, Silvia G. Priori, Sakis Themistoclakis, Matteo Anselmino, Roberto De Ponti, on behalf of the Italian Association of Arrhythmology and Cardiac Pacing (AIAC)

**Affiliations:** ^1^Division of Cardiology, Umberto Parini Regional Hospital, 11100 Aosta, Italy; ^2^Division of Cardiology, Ospedale di Rivoli, 10098 Rivoli, Italy; ^3^Division of Cardiology, Giovanni Paolo II Hospital, 07026 Olbia, Italy; ^4^Struttura Complessa Cardiologia, Ospedale Maria Vittoria-Martini, 10153 Torino, Italy; ^5^Unità Operativa Semplice Terapia Intensiva Cardiologica, Azienda Ospedaliero-Universitaria Policlinico Paolo Giaccone, 90127 Palermo, Italy; ^6^Unità Operativa Complessa Cardiologia—Unità di Terapia Intensiva Cardiologica, Presidio Ospedaliero Rossano Calabro, 87067 Rossano Calabro, Italy; ^7^Division of Interventional Electrophysiology, Fondazione Toscana Gabriele Monasterio, 56124 Pisa, Italy; ^8^Struttura Complessa di Cardiologia Universitaria, Azienda Ospedaliero-Universitaria Policlinico Riuniti, 71122 Foggia, Italy; ^9^Unità Operativa Complessa di Cardiologia, Ospedale San Bortolo, 36100 Vicenza, Italy; ^10^Institute of Cardiology, IRCCS Azienda Ospedaliero-Universitaria di Bologna, 40138 Bologna, Italy; ^11^Cardiology Division, Department of Biomedical, Metabolic and Neural Sciences, University of Modena and Reggio Emilia, Policlinico di Modena, 41124 Modena, Italy; ^12^Cardiologia-Elettrofisiologia, Clinica Montevergine, 83040 Mercogliano (AV), Italy; ^13^Cardiologia Molecolare, Istituti Clinici Scientifici Maugeri, IRCCS, 27100 Pavia, Italy; ^14^Unità Operativa Complessa di Cardiologia, Ospedale Dell’Angelo, 30174 Mestre-Venice, Italy; ^15^Division of Cardiology, Cardiovascular and Thoracic Department, “Città della Salute e della Scienza” Hospital, 10126 Turin, Italy; ^16^Department of Medical Sciences, University of Turin, 10126 Turin, Italy; ^17^Department of Medicine and Surgery, University of Insubria, 21100 Varese, Italy

**Keywords:** atrial fibrillation, catheter ablation, procedural sedation, general anesthesia, ablation techniques

## Abstract

Catheter ablation is the mainstay of rhythm control in atrial fibrillation (AF), and its use is steadily increasing worldwide. To optimize procedural safety and efficacy, an appropriate sedation or anesthesia regimen is essential, ensuring adequate analgesia and a stable respiratory pattern while minimizing patient movement. However, the optimal sedation strategy remains a matter of debate, with approaches ranging from general anesthesia to deep or conscious sedation. Since anesthesiologists administer general anesthesia, attention focuses on hypnotics, defined as drugs that induce and/or maintain sleep by depressing the central nervous system, and analgesics. In many centers, these agents are administered by electrophysiology laboratory staff in accordance with local regulations and institutional protocols, which vary among countries. This warrants caution, as individual responses to commonly combined agents are unpredictable and may result in deeper-than-intended sedation. Therefore, respiratory or hemodynamic support may become necessary in selected patients. Notably, protocols incorporating hypnotic communication have also been proposed and implemented. The selection of sedation and analgesia strategies for AF ablation has become increasingly important for balancing patient safety, procedural effectiveness, and resource utilization. The approach should be individualized based on patient characteristics, procedural complexity, energy source, institutional resources, and the relevant national regulatory framework.

## 1. Introduction

Catheter ablation of atrial fibrillation (AF) is an established therapy for rhythm control in affected patients [1, 2, 3]. In the general population with symptomatic paroxysmal or persistent AF, catheter ablation reduces arrhythmia burden and improves quality of life, while in selected patients with heart failure with reduced ejection fraction, catheter ablation improves major clinical outcomes, including mortality, by significantly reducing recurrences [2]. The 2024 European Society of Cardiology guidelines [2, 3] recommend catheter ablation as a first-line treatment option, based on shared decision-making, in patients with paroxysmal AF to reduce symptoms, recurrences, and progression of the arrhythmia; consequently, the number of candidates for this procedure is increasing worldwide. Moreover, in a simulated model of the Italian and French healthcare systems, early catheter ablation potentially increases the proportion of patients with controlled AF and reduced adverse events, offsetting the modest increase in total AF-related costs [4].

Pulmonary vein isolation (PVI) remains the cornerstone of AF ablation [1]. Over time, advances in knowledge, operator experience, and technology have reduced both procedure duration and ablation time [5, 6]. Conversely, significant structural heart disease, concomitant arrhythmias such as atrial flutter or atrial tachycardia [7, 8], and/or the need for additional ablation beyond PVI in patients with persistent AF [9] are associated with longer procedures. Therefore, appropriate periprocedural management is essential, particularly because pain, respiratory variability, and body movement may influence clinical outcomes, catheter stability, and the accuracy of three-dimensional mapping. Specifically, as in several centers, three-dimensional mapping is used to support AF ablation and decrease radiation exposure [5]. The adoption of appropriate sedation strategies is crucial, as patient movement compromises the reliability of catheter visualization during electroanatomic mapping and disrupts procedural workflow.

In this context, sedation and anesthesia strategies during AF ablation have never been strictly standardized, and the optimal modality remains debated. A recent international consensus document [1] states that the procedure can be performed under general anesthesia, deep sedation, or conscious sedation, depending on patient and procedural characteristics, operator experience, anesthesia availability, and institutional protocols, and recommends a multidisciplinary approach. Accordingly, worldwide practice is heterogeneous and influenced by patient characteristics, regulatory constraints, availability of dedicated personnel, and monitoring technologies. A global survey promoted by the European Heart Rhythm Association [10] showed that, in 2019, general anesthesia was the most frequently used strategy (40.5%), followed by conscious sedation (32.0%) and deep sedation (27.5%), with marked geographical and volume-related variability. General anesthesia predominated in North and South America, whereas conscious or deep sedation was more common in Europe, although variability among European countries persisted. Between 2010 and 2019, low-volume centers increased the use of deep sedation and decreased the use of conscious sedation, while high-volume centers increased the use of general anesthesia and decreased the use of conscious sedation. Propofol and midazolam were the most commonly used hypnotic agents, and remifentanil and fentanyl were the most frequently used analgesics. In 2021, the Italian Association of Arrhythmology and Cardiac Pacing reported the results of a national survey on anesthesiology practice in interventional electrophysiology and cardiac pacing [11]. During AF ablation, approximately half of the centers used deep sedation or general anesthesia with an anesthesiologist, 40% administered sedation without an anesthesiologist, and 10% used local anesthesia alone.

The most critical phase of AF ablation is energy delivery, as different energy sources are associated with varying degrees of pain perception. When radiofrequency (RF) or cryothermal energy is used, similar sedation protocols are adopted in most centers [10]. However, the recent introduction and increasing use of pulse-field ablation (PFA), which is expected to be less well tolerated due to procedure-related pain [12], have added another variable. This energy source is associated with increased requirements for sedative and analgesic agents [13]. Although in a large international registry, the most common strategy for PFA was general anesthesia or deep sedation with continuous propofol infusion [14], smaller series of younger patients have demonstrated satisfactory results using alternative regimens combining hypnotics and opioids while avoiding propofol because of the risk of respiratory depression, without the need for noninvasive ventilation or orotracheal intubation [15, 16].

Therefore, anesthetic management requires a careful balance among analgesia, prolonged immobility, respiratory depression, and hemodynamic effects. Deeper sedation improves catheter stability and lesion formation and durability, and invasive airway management stabilizes respiratory cycles by preventing alternating apnea and deep breathing. Conversely, invasive airway management prolongs laboratory occupancy time and may increase complications in selected patients. Robust evidence identifying the optimal strategy remains lacking. However, a meta-analysis of randomized and observational studies of RF ablation demonstrated a trend toward lower AF recurrence with general anesthesia or deep sedation, without significant differences in procedural parameters or overall complications; most complications in the general anesthesia/deep sedation group were related to intubation or anesthesia [17]. Nonetheless, due to heterogeneity across studies, definitive conclusions could not be drawn. A more recent meta- analysis including 15 observational studies and 6 randomized trials found no significant differences in post-ablation recurrence between general anesthesia and sedation [18]. Although RF was the predominant energy source, studies using PFA and cryoballoon ablation were also included.

This review aims to describe the pharmacologic characteristics of commonly used agents, summarize the available literature on sedation protocols, and provide an overview of regulatory requirements for sedation and general anesthesia, which are crucial in this multidisciplinary setting.

## 2. Definition of General Anesthesia, Deep Sedation, and Conscious Sedation

In 1999, the American Society of Anesthesiologists defined levels of sedation and analgesia as distinct from general anesthesia, emphasizing that sedation exists on a continuum and that individual patient responses cannot always be predicted. Consequently, the depth of sedation may fluctuate at any point during a procedure, requiring prompt recognition and appropriate corrective measures. The most recent update of this document was published in October 2024 [19]. Excluding minimal sedation, which essentially provides anxiolysis and is generally inadequate for AF ablation procedures, interventions are usually performed under conscious sedation, deep sedation, or general anesthesia. Both conscious and deep sedation are typically achieved through titrated intravenous administration of sedative and/or analgesic agents throughout the procedure. However, during deep sedation, individual variability and the use of drug combinations may impair the ability of the patient to maintain spontaneous ventilation. Thus, ventilatory function may become inadequate, and airway support may be required to maintain airway patency. For this reason, practitioners administering any level of sedation must be able to promptly rescue patients who inadvertently progress to a deeper level of sedation or general anesthesia, using effective airway management and advanced life support measures [19].

During AF ablation, general anesthesia requires airway control with an endotracheal tube or a laryngeal mask [20], which, in certain organizational settings, may prolong the overall procedural duration. A recent retrospective single-center study reported a 42.2% incidence of respiratory complications in patients undergoing AF ablation with RF or cryoablation under sedation/analgesia [21]. When subdivided into mild, intermediate, or severe according to the Tracking and Reporting Outcomes of Procedural Sedation tool, complications occurred in 0.4%, 35%, and 15.1% of cases, respectively. Longer procedure duration, age >50 years, neck circumference >40 cm, increased visceral fat percentage, and a history of obstructive sleep apnea syndrome or chronic obstructive pulmonary disease were among the main risk factors for periprocedural respiratory complications.

## 3. Medications

The main pharmacological properties of drugs commonly used during AF ablation are summarized in Table 1.

**Table 1.** **Hypnotic and analgesic agents used in atrial fibrillation (AF) ablation**.

Agents	Hypnotic properties	Analgesic properties	Effect onset	Effect duration	Main side effects	Antagonist
Midazolam (intravenous administration)	+++	+	∼3 minutes	∼2–3 hours	Respiratory depression	Yes, flumazenil
Propofol	+++	no	30–40 seconds	5–10 minutes	Respiratory depression	No
					Hypotension	
					Negative inotropic effect	
Dexmedetomidine	+++	+	∼5 minutes	2–3 hours	Hypotension	No
					Bradycardia	
					Vasoconstriction	
Fentanyl/Remifentanil	No	+++	Fentanyl: 5 minutes	Fentanyl: 30–60 minutes	Respiratory depression	Yes, naloxone
			Remifentanil: 1–2 minutes	Remifentanil: 3–10 minutes	Increased vagal tone (bradycardia, hypotension)	
Ketamine	++	++	30–60 seconds	2–3 hours	Dissociation and hallucinations	No
					Increase sympathetic tone (tachycardia, hypertension)	
Paracetamol/Acetaminophen	No	+	30 minutes	4–6 hours	No major side effects	No

### 3.1 Hypnotic Agents

#### 3.1.1 Midazolam

Midazolam is a benzodiazepine hypnotic that induces and maintains sleep through central nervous system depression. Benzodiazepine-based conscious sedation reduces anxiety and provides amnesia and mild analgesia during minor procedures. The pharmacologic effects of midazolam are similar to those of other benzodiazepines and include sedation, anxiolysis, sleep induction, and amnesia; however, this medication has a faster onset and shorter duration of action due to the associated high lipophilicity. The onset of action occurs approximately 3 minutes after intravenous administration and 9–26 minutes after oral administration [22]. Midazolam has been associated with respiratory depression, cardiac arrest, and death, particularly when combined with opioids, although the incidence of severe adverse events is low. Flumazenil effectively reverses benzodiazepine-induced sedation; however, respiratory depression may persist and only rarely requires assisted ventilation [22]. Conscious sedation with midazolam, followed by flumazenil and administered by a cardiologist during electrical cardioversion of AF, is safe, effective, and well tolerated, facilitating the procedure and shortening its duration [23]. This approach has been extrapolated to AF ablation, typically in combination with opioids to optimize analgesia and maintain patient immobility [24]. Administration is usually performed using repeated intravenous boluses [25], although continuous infusion has also been described [26].

#### 3.1.2 Propofol

Propofol is an intravenous anesthetic agent that primarily acts as a positive allosteric modulator of the GABA receptor. Propofol is highly lipophilic, has rapid tissue distribution, and undergoes fast metabolic clearance, which facilitates rapid penetration of the blood–brain barrier and results in an onset of action within 30–40 seconds and rapid recovery [27]. Propofol may be administered as intermittent boluses, continuous infusion, or a combination of both. In prolonged procedures such as AF ablation, continuous infusion helps maintain more stable levels of sedation and reduces fluctuations in arousal [27]. Propofol produces significant hypotension and, particularly in patients with heart failure, exerts negative inotropic effects that may necessitate the supervision of an anesthesiologist in selected high-risk patients [28]. Increased body mass index (BMI), higher propofol dose, persistent AF, longer procedural duration, and obstructive sleep apnea have all been identified as predictors of the need for non-invasive ventilation during propofol sedation [29, 30]. Moreover, because propofol lacks intrinsic analgesic properties, concomitant administration of analgesics is generally required [27].

Propofol is listed among drugs that should possibly be avoided in patients with Brugada syndrome, a population not infrequently undergoing AF ablation, as AF is part of the clinical manifestations of the syndrome. Although the evidence remains conflicting, available data suggest a possible proarrhythmic effect [31].

#### 3.1.3 Dexmedetomidine

Dexmedetomidine is a highly selective α2-adrenergic agonist with sedative and anxiolytic properties, provides analgesia, attenuates stress responses, and is widely used in intensive care units and during endoscopic or minor surgical procedures [32]. Activation of central and peripheral α2-adrenergic receptors inhibits norepinephrine release and sympathetic activity, leading to hypotension and bradycardia, whereas activation of spinal α2-receptors produces analgesia [32, 33]. Dexmedetomidine is rapidly distributed to tissues, with a distribution half-life of approximately 5 minutes and an elimination half-life of 2–3 hours. Dexmedetomidine is metabolized in the liver and, therefore, requires caution in patients with severe liver insufficiency, whereas no dose adjustment is required in renal dysfunction. Elimination occurs via both urinary and fecal excretion [32]. Importantly, therapeutic doses generally do not produce clinically significant respiratory depression compared with benzodiazepines or opioids [32, 33]. A rare but potentially serious adverse effect is coronary vasospasm, reported in an isolated case [34]. Since α2-receptor stimulation can induce vascular smooth muscle contraction, rapid administration may cause transient hypertension and vasoconstriction, particularly in patients with endothelial dysfunction or atherosclerosis [34].

### 3.2 Analgesic Agents

#### 3.2.1 Opioid Analgesics

Fentanyl is a synthetic piperidine µ-opioid receptor agonist with a rapid onset, achieving maximal analgesia within approximately 5 minutes, and a short duration of action of 30–60 minutes [35]. Fentanyl is metabolized by CYP3A4 and cleared by the kidneys; therefore, dose adjustment is required in patients with hepatic or renal impairment.

Remifentanil is a selective µ-opioid receptor agonist with a rapid onset and an ultra-short duration of action, characterized by an effective biological half-life of 3–10 minutes. Remifentanil is rapidly hydrolyzed by plasma and tissue esterases. Moreover, since remifentanil may inhibit sinus and atrioventricular nodal activity, bradycardia may occur [35]. Particularly in older patients, remifentanil may induce chest wall rigidity (the so-called “wooden chest syndrome”), resulting in ineffective ventilation, hypoxemia, hypercarbia, and desaturation [36]. Pretreatment with hypnotics mitigates this risk [36]. Both fentanyl and remifentanil produce significant respiratory depression and increase vagal tone; the effects of these agents can be antagonized with specific opioid antagonists, such as naloxone. However, abrupt reversal may provoke marked sympathetic activation and increase the risk of ventricular arrhythmias, including sustained ventricular tachycardia or ventricular fibrillation [37].

Sufentanil is a synthetic opioid, structurally related to fentanyl but more potent due to stronger affinity for the µ-opioid receptors and higher lipophilicity, which facilitates rapid central nervous system penetration and onset of action [38].

Morphine is a hydrophilic natural opioid with slower central nervous system penetration due to the blood–brain barrier, and an onset of action within a few minutes. Similar to other opioids, sufentanil and morphine may cause respiratory depression, even in the postoperative phase [38], as well as nausea and vomiting [39]. Both agents are infrequently used during AF ablation [16, 24].

#### 3.2.2 Paracetamol/Acetaminophen

Proactive management of post-procedural pain is integral to the AF ablation procedure, as moderate-to-severe pain within the first 24 hours after ablation is frequently reported [40]. Nonetheless, optimal strategies for post-procedural analgesia remain undefined. N-acetyl-para-aminophenol, known as paracetamol in Europe and acetaminophen in the United States, was first synthesized in 1878 [41]. The antipyretic and analgesic effects of N-acetyl-para-aminophenol were initially attributed to inhibition of prostaglandin synthesis via the cyclooxygenase pathway. However, more recent evidence suggests that active metabolites interact with cannabinoid receptors, elevating endogenous cannabinoid levels and thereby contributing to analgesia [41, 42]. Unlike other non-steroidal anti-inflammatory drugs, paracetamol has limited anti-inflammatory activity, minimal gastrointestinal or cardio-renal toxicity, and no respiratory depressant effects [42], making this medication suitable for post-procedural pain management. In fact, paracetamol has demonstrated significant analgesic efficacy and opioid-sparing effects after both cardiac and non-cardiac surgery, and enhanced recovery protocols recommend scheduled rather than as-needed administration [43]. In adults, typical oral doses range from 650 to 1000 mg every 6–8 hours, with a maximum daily dose of 3 g. Onset of analgesia occurs approximately 30 minutes after oral administration in fasting subjects, with a duration of about 4 hours [42]. Nevertheless, because paracetamol is hepatically metabolized, caution is warranted in patients with liver insufficiency [43].

### 3.3 Analgesic and Hypnotic Agents: Ketamine

Ketamine is an N-methyl-D-aspartic acid (NMDA) receptor antagonist with combined sedative and analgesic properties [44]. Analgesia is achieved by inhibiting NMDA receptors, resulting in central nervous system depression and dissociation. Following intravenous administration, peak plasma concentrations are reached within 2–4 minutes, and high lipid solubility enables rapid penetration of the blood–brain barrier. Ketamine has a half-life of 2–3 hours, and the clearance of this drug is largely independent of renal function. Ketamine uniquely provides both sedation and analgesia while preserving hemodynamic and respiratory reflexes.

Ketamine also has sympathomimetic effects, mediated by the release of endogenous catecholamines, inhibition of vagal activity, and inhibition of norepinephrine reuptake, resulting in increased heart rate and blood pressure, as well as potential arrhythmogenicity. A well-known adverse effect is the emergence reaction, which occurs in up to 30% of patients and is characterized by psychomotor disturbances during recovery; this may lead to detrimental patient movement during procedures [44].

Experience with ketamine in AF ablation is limited [16, 45]. Intravenous ketamine, administered after premedication with midazolam and fentanyl approximately 5 minutes before PFA, with titration to a total dose of 3 mg/kg, has been reported without major procedural or anesthesia-related complications [45].

## 4. Alternative Strategies

Hypnosis is increasingly recognized as an effective approach for managing acute and chronic pain. Hypnosis involves a modified state of consciousness characterized by reduced self-awareness and increased responsiveness to suggestions [46]. Multiple studies have demonstrated the effectiveness of hypnosis in chronic pain management [47, 48] and in reducing procedural pain [49, 50]. Indeed, when used as an adjunct to conventional analgesia, hypnosis can enhance analgesic effectiveness and decrease sedative and opioid requirements. In 2019, Scaglione *et al*. [51] conducted an observational study comparing hypnotic communication with conventional analgesia in two cohorts of 70 patients undergoing RF ablation of AF. Hypnosis induction was successfully achieved in 97% of cases. Compared with conventional analgesia, the hypnosis group experienced significant reductions in procedural anxiety and perceived procedural duration. Procedural pain was reported as absent in 78% of patients, and no patient required invasive or non-invasive ventilatory support or additional major sedatives (midazolam or propofol). Fentanyl doses were similar in both groups, whereas higher doses of paracetamol were required in the hypnosis cohort. No differences were observed in procedural safety, acute success rates, or total RF and fluoroscopy times. These findings are promising, although replication in larger cohorts is required. Visualization, a technique in which patients are guided to modify associated subjective experience, thoughts, or behavior, has also been reported to reduce procedural pain and analgesic requirements during AF ablation [52].

## 5. Drug Interactions and Side Effects of Drug Administration and Energy Delivery

A thorough pre-procedural assessment of chronic medication use is essential to minimize drug–drug interactions during anesthesia, particularly with psychotropic agents. Antidepressants (tricyclics, selective serotonin reuptake inhibitors, serotonin–norepinephrine reuptake inhibitors), antipsychotics, anxiolytics, and stimulants can modify anesthetic effects, either enhancing or, more commonly, reducing efficacy, and may precipitate serious side effects [53].

Patients with chronic alcohol use disorder should be identified pre-procedurally, and analgesic and sedative regimens adjusted accordingly, including opioid dose reduction and careful benzodiazepine titration [54]. These patients have an increased risk of postoperative complications such as bleeding and delirium and require close monitoring [54].

Postoperative nausea and vomiting are common after anesthesia. Current guidelines recommend pre-procedural risk assessment and prophylactic pharmacologic interventions, including ondansetron, droperidol, and dexamethasone [55].

PFA may stimulate the phrenic nerve and bronchus, leading to diaphragmatic contraction and dry cough, particularly during left superior PVI [56]. These events can induce patient movement and compromise the accuracy of three-dimensional mapping. Delivering PFA at the end of expiration can reduce diaphragmatic contraction and suppress dry cough during procedures performed under conscious sedation [57]. Although no studies have specifically evaluated intravenous lidocaine to prevent cough during PFA, many anesthesiologists advocate the use of intravenous lidocaine based on evidence supporting postoperative cough reduction [58]. Finally, vagal responses may also occur during PFA; prophylactic atropine administration has been shown to mitigate this effect [56].

## 6. Protocol for Deep and Conscious Sedation

While general anesthesia is strictly protocolized and administered by anesthesiologists, the pharmacological agents previously described have been employed in heterogeneous combinations, either as continuous intravenous infusions or as intermittent boluses, to achieve deep or conscious sedation. A dedicated scoping review providing a structured overview of anesthetic strategies for cardiac ablation, particularly AF, is currently underway [59]. Meanwhile, Table 2 (Ref. [13, 15, 16, 27, 30, 33, 60, 61]) and Table 3 (Ref. [20, 25, 26, 60, 62]) summarize the principal protocols used for deep and conscious sedation, respectively.

**Table 2.** **Protocols for deep sedation used in AF ablation**.

Author and year	Number of patients	Energy source	Inclusion/exclusion criteria	Drug	Need for NIV or OTI	Sedation-related side effects
Salukhe *et al*. 2012 [27]	1000	RF	All consecutive patients	Propofol (2%)	None	Persistent hypotension (13.6%)
				Fentanyl		Respiratory depression (1.9%)
						Hypersalivation (0.1%)
Cho *et al*. 2014 [60]	45	RF	Excluded patients with ASA score ≥III, respiratory disease, and end-stage renal disease	Dexmedetomidine + remifentanil	None	Hypotension (11.1%)
Foerschner *et al*. 2022 [30]	3211	RF	All consecutive patients	Midazolam	NIV (1.5%)	Persistent hypotension (12.3%)
				Propofol (1%)	OTI (0.03%)	
				Fentanyl		
Servatius *et al*. 2022 [33]	160	RF (50%)	Excluded patients with severe HF, BMI >35, ASA class >III	Dexmedetomidine + fentanyl vs. propofol + fentanyl	None	Prolonged hypotension (8%)
		Cryo (50%)				
Grimaldi *et al*. 2023 [15]	29	PFA	All consecutive patients	Midazolam	None	None
				Dexmedetomidine		
				Remifentanil		
				(premedication with ondansetron and dexamethasone)		
Iacopino *et al*. 2023 [16]	66	PFA	All consecutive patients	Midazolam	None	Vomit after fentanyl (1.5%)
				Fentanyl		
				Ketamine		
Wahedi *et al*. 2024 [13]	100	Cryo (50%)	Redo‐ablation procedures excluded	Midazolam	None	Aspiration resulting in mild pneumonia (2%)
		PFA (50%)		Propofol (1%)		
				Sufentanil		
Rillig *et al*. 2024 [61]	63	PFA	Redo‐ablation procedures excluded	Fentanyl	Larynx mask (2.5%)	None
				Propofol (1%)		

Abbreviations: NIV, non-invasive ventilation; OTI, orotracheal intubation; RF, radiofrequency energy ablation; Cryo, cryoablation; PFA, pulsed-field ablation; HF, heart failure; BMI, body mass index; ASA, American Society of Anesthesiologists.

**Table 3.** **Protocols for conscious sedation used in AF ablation**.

Author and year	Number of patients	Energy source	Inclusion/exclusion criteria	Drug	Need for NIV/OTI	Sedation-related side effects
Cho* et al*. 2014 [60]	45	RF	Excluded patients with ASA score ≥III, respiratory disease, and end-stage renal disease	Midazolam Remifentanil	None	Oxygen saturation <90% (33%)
Moravec *et al*. 2021 [26]	73	RF	Excluded patients with persistent AF, LVEF <35%	Midazolam Fentanyl	None	None
Calvert *et al*. 2025 [25]	8	PFA	Excluded patients with BMI >40, obstructive sleep apnea or other airway concerns, or undergoing posterior wall isolation	Midazolam Fentanyl	None	None
Poggi *et al*. 2025 [62]	58	RF with vHPSD protocol	Excluded redo‐ablation procedures	Midazolam Fentanyl	None	None
Massalha *et al*. 2025 [20]	399	Cryo 88%	N/A	Midazolam Fentanyl	None	None
		RF 12%				

Abbreviations: LVEF, left ventricular ejection fraction; vHPSD, very-high-power, short duration; N/A, not available; other abbreviations as in Table 2.

### 6.1 Deep Sedation

Deep sedation is generally achieved by combining hypnotic and analgesic agents (*e*.*g*., propofol with fentanyl or dexmedetomidine with remifentanil), titrated individually to the desired level of sedation.

In a large study by Salukhe* et al*. [27], 1000 consecutive patients underwent AF ablation under deep sedation without assisted ventilation using 2% propofol and fentanyl. Sedation was administered, monitored, and controlled entirely by electrophysiologists. Sedation-related adverse effects requiring discontinuation of propofol and conversion to midazolam boluses occurred in 15.6% of patients (13.6% due to persistent hypotension, 1.9% due to respiratory depression, and 0.1% due to hypersalivation). Only one patient required brief (4 minutes) mechanical bag-mask ventilation, and no procedures were aborted because of sedation-related complications.

In a randomized controlled trial by Cho *et al*. [60], 45 patients receiving dexmedetomidine plus remifentanil were compared with those receiving midazolam plus remifentanil. Patients in the dexmedetomidine group achieved a median Ramsay sedation score of 3 and a bispectral index of 70, consistent with deep sedation. Dexmedetomidine was administered at 0.2–0.7 µg/kg/h following a loading dose of 1.0 µg/kg over 10 minutes, and remifentanil was infused at 1.2–2.4 µg/kg/h. No invasive airway management was required; five patients experienced isolated hypotensive episodes, which were corrected by adjusting infusion rates. Compared with the midazolam group, dexmedetomidine was associated with less respiratory depression, deeper sedation, improved analgesia, and lower remifentanil requirements.

In 2022, Foerschner *et al*. [30] retrospectively analyzed 3211 consecutive AF ablations performed under deep sedation. Sedation was initiated with midazolam and propofol boluses, followed by a continuous infusion of 1% propofol administered by an electrophysiology nurse under physician supervision, with supplemental fentanyl boluses for discomfort or pain. Hypotension occurred in 12.3% of patients and was treated with norepinephrine. Only one patient (0.03%) required intubation, while 47 patients (1.5%) required non-invasive ventilation due to oxygen saturation <85%. In multivariable analysis, BMI >30 kg/m^2^ was the sole independent predictor of ventilation requirement.

In a single-center randomized trial, Servatius *et al*. [33] enrolled 160 patients undergoing initial AF ablation (RF or cryoablation) and randomized the patients to receive either dexmedetomidine or propofol. Deep sedation was administered by a trained nurse under the operator’s supervision. Propofol was delivered via target-controlled infusion (a technique that uses a computer-controlled infusion pump that delivers the drug based on patient-specific parameters to achieve a predicted plasma concentration). In contrast, dexmedetomidine was infused continuously (0.2–0.7 µg/kg/h) following a loading dose (0.5–1 µg/kg over 10 minutes), with adjunctive fentanyl boluses. Hypercapnia was more frequent with propofol (29% vs. 10%; *p* = 0.003), whereas the incidence of prolonged hypotension did not differ significantly between groups (3% vs. 8%; *p* = 0.147). Patient satisfaction was higher in the propofol group.

More recently, deep sedation has also been investigated for PFA. In a study by Grimaldi *et al*. [15], patients received pre-procedural intravenous midazolam 2 mg, ondansetron 4 mg, and dexamethasone 4 mg. The degree of sedation was subsequently assessed using the Richmond agitation–sedation scale, the visual analog scale, and the patient state index. During ablation, dexmedetomidine (0.5–1.4 µg/kg/h) and remifentanil (target-controlled infusion 0.5–2 ng/mL) were administered by electrophysiology-trained staff and titrated to achieve a patient state index score <50, and a visual analog scale score of 0–2. No significant hypotension or intubation occurred.

Iacopino *et al*. [16] described a ketamine-based deep sedation protocol with spontaneous respiration for PFA, managed by an anesthesiologist and an operating room nurse. Patients received midazolam (2 mg) and fentanyl (1.5 µg/kg), followed by local anesthesia. An additional midazolam bolus (2 mg) was administered after transseptal puncture, followed by ketamine (1 mg/kg) administered 5 minutes before energy delivery and titrated to the condition, response, vital signs, and cough of the patient. Maintenance ketamine boluses of 10 mg were administered once the effective anesthetic level was reached. Mean sedation time was 56.4 ± 6 minutes, and no major complications occurred. In one case, the sedation strategy was modified due to fentanyl-related nausea and vomiting.

Wahedi *et al*. [13] compared a sedation regimen that included midazolam, a propofol bolus and infusion, and sufentanil boluses between patients undergoing PFA and cryoablation. Sedative drug requirements were significantly higher in the PFA group, whereas complication rates were similar, with only one mild case of aspiration pneumonia in the PFA group.

Rillig *et al*. [61] compared deep sedation with general anesthesia for PFA. Deep sedation was managed by trained electrophysiology laboratory staff and consisted of fentanyl (25 µg) and propofol (3–5 mg) boluses, followed by a propofol infusion (5 mg/kg/h), with additional boluses given before ablation onset and as required thereafter. Only one patient with BMI >40 kg/m^2^ required conversion to laryngeal mask ventilation.

Respiratory depression represents the most common adverse event during deep sedation. Capnography enables monitoring of airway patency, respiratory rate and pattern, and pulmonary perfusion. Both the European and American Societies of Anesthesiology recommend using capnography, in addition to visual observation and pulse oximetry, for the early detection of ventilatory impairment [63, 64]. Capnography uses infrared spectroscopy to measure end-tidal CO_2_ (ETCO_2_); changes in ETCO_2_ values or waveform morphology enable early detection of hypoventilation or apnea, which typically precede hypoxemia.

### 6.2 Conscious Sedation

In the midazolam–remifentanil arm of the study by Cho *et al*. [60], patients achieved a median Ramsay score of 2 and a bispectral index score of 90, consistent with conscious sedation. Sedation was provided using midazolam 0.02–0.05 mg/kg as a bolus and remifentanil infusion (3.6–7.2 µg/kg/h). Desaturation occurred in 33% of patients and was managed with supplemental oxygen via a face mask and airway maneuvers (head elevation, chin lift, jaw thrust); no invasive airway support was required.

Moravec *et al*. [26] compared 73 patients undergoing RF ablation under conscious sedation with 73 patients undergoing RF ablation under general anesthesia. Conscious sedation consisted of fentanyl 100 µg as an intravenous bolus and a continuous midazolam infusion (initiated at 0.03–0.2 mg/kg/h and titrated to achieve the target level of sedation according to the Richmond agitation–sedation scale: –2 to –3). No sedation-related complications were observed. Clinical outcomes were comparable to those under general anesthesia, although procedure duration and fluoroscopy time were longer in the conscious sedation group.

In a small PFA series, Calvert *et al*. [25] administered midazolam (1–2 mg) and fentanyl (50 µg) as boluses at the beginning of the procedure, before the first ablation delivery, with additional doses administered at the discretion of the operator. One patient required conversion to general anesthesia due to pain. No respiratory depression or hypotension requiring intervention was reported.

Poggi *et al*. [62] evaluated conscious sedation during very-high-power, short-duration RF ablation (90 W, 4 s lesions, temperature-controlled protocol). Midazolam 1–2 mg, followed by fentanyl 50–100 µg, was administered upon request to achieve pain control. Only 7% of patients required fentanyl, and approximately 40% underwent ablation without any anesthetic drugs.

Finally, the Israeli Catheter Ablation Registry, analyzed by Massalha *et al*. [20], included 1002 patients: 53% received general anesthesia, 6.3% deep sedation, and 40% conscious sedation (midazolam and fentanyl administered by electrophysiologists or trained cardiology nursing staff). In this registry, rates of PVI achievement, complications, and AF recurrence at 2-year follow-up did not differ between general anesthesia and conscious sedation. Predictors of general anesthesia use included RF energy, high-volume centers, persistent AF, and prior cerebrovascular events.

## 7. Regulatory Framework and Organizational Model

In several studies, drug administration and monitoring were performed by specially trained electrophysiology staff. However, drug administration may still be associated with respiratory depression and persistent hypotension [13, 27, 30, 33, 60], especially when deep sedation is achieved (Table 2), and occasionally requires non-invasive ventilation, a laryngeal mask airway, or endotracheal intubation [30, 61]. Therefore, as recommended by the American Society of Anesthesiologists [19], practitioners administering sedation must be able to rescue patients whose level of sedation becomes deeper than intended.

In the European Heart Rhythm Association survey [10], national legislation governing procedural sedation was reported to vary considerably across European countries. In some jurisdictions, including France, Spain, and Italy, deep sedation with specific agents cannot legally be performed without an anesthesiologist present in the operating room/interventional laboratory [65]. Conversely, in countries such as Germany, standardized protocols allow deep sedation with propofol during AF ablation without an anesthesiologist in attendance. In the same survey, 60% of centers reported that an anesthesiologist was required in the electrophysiology laboratory. Another 29% indicated that deep sedation could be administered in the presence of a specifically trained nurse or a second physician, whereas 11% reported that electrophysiologists could independently administer deep sedation. Similarly, in an Italian survey on deep sedation practices in electrophysiology and cardiac pacing laboratories [11], AF ablation was most commonly performed with anesthesiology assistance or with benzodiazepine-based sedation alone. However, in a minority of centers (19%), propofol was administered without anesthesiology support, presumably within predefined and institutionally approved protocols. These findings likely reflect the organizational structure of the participating institutions, as only 27% were high-volume centers performing more than 100 procedures annually. This scenario may evolve with increasing procedural volumes and the progressive implementation of structured organizational models, as observed worldwide [10]. Furthermore, the Italian consensus document on quality and performance in cardiac pacing and electrophysiology, issued by the Italian Association of Arrhythmology and Cardiac Pacing together with the Italian Federation of Cardiology, requires that an anesthesiologist be available at tertiary centers performing left-heart ablation procedures [66]. Notably, the literature on AF catheter ablation lacks recent, comprehensive, and standardized training protocols for the administration of conscious or deep sedation by nurses or non-anesthesiologist physicians, representing an important area for future investigation.

**Fig. 1.**
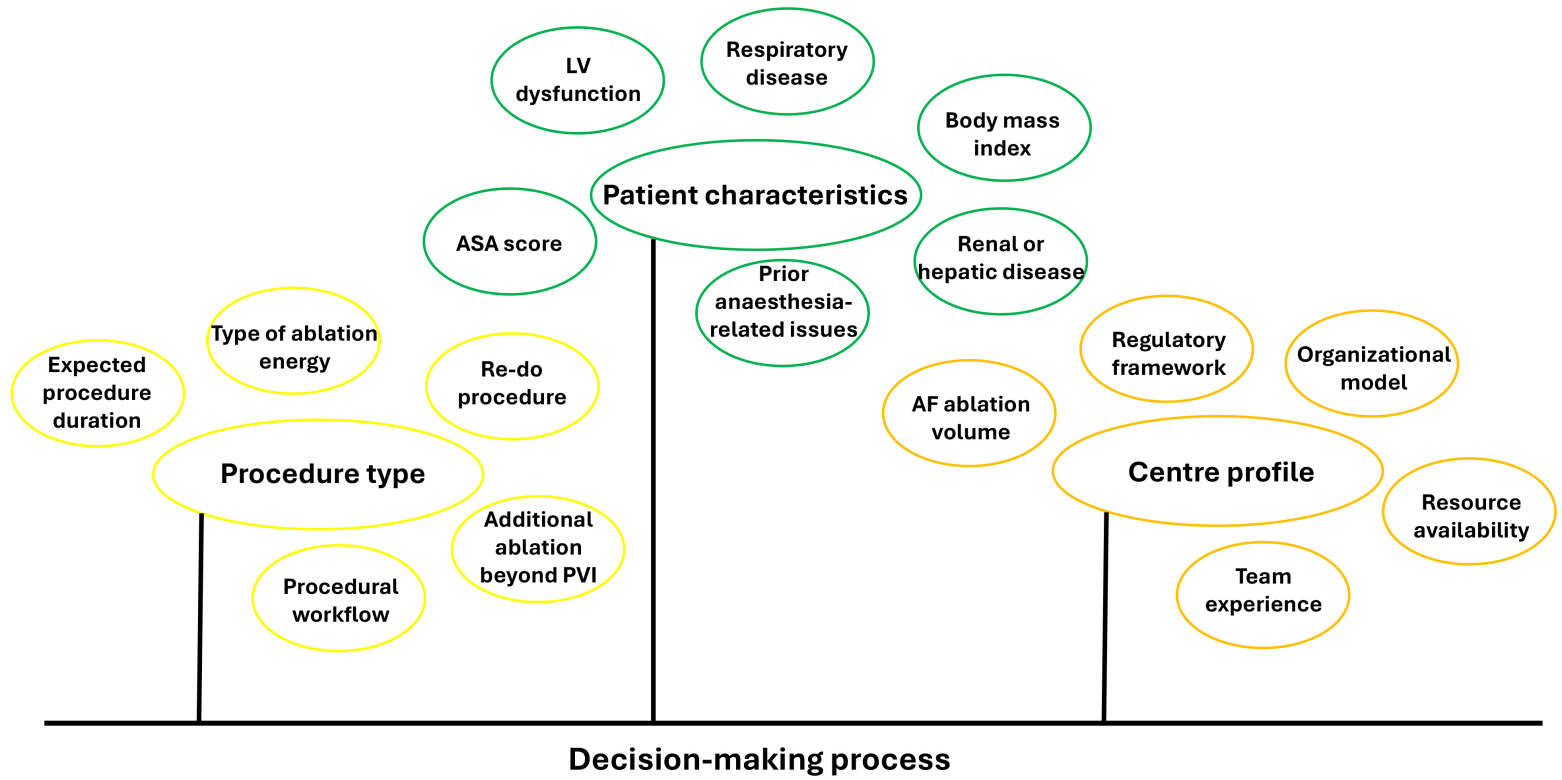
**Factors to consider in the selection of a sedation and analgesia strategy for patients undergoing atrial fibrillation ablation**. Abbreviations: AF, atrial fibrillation; ASA, American Society of Anesthesiologists; LV, left ventricular; PVI, pulmonary vein isolation.

Within this framework, sedation strategies should be tailored according to patient characteristics, energy source, expected procedural duration, and anticipated pain burden [67]. Optimal patient management requires predefined, shared protocols between electrophysiologists and anesthesiologists, supported by an appropriate organizational model. As illustrated in Fig. 1, the choice among general anesthesia, deep sedation, and conscious sedation should be based on multidisciplinary decision-making that incorporates patient profile, procedural complexity, and institutional expertise, with important implications for clinical outcomes, costs, and resource utilization.

## 8. Conclusions

The number of AF ablation procedures is increasing worldwide as clinical indications expand. Accordingly, the choice of sedation–analgesia strategy has become a key determinant of patient safety and comfort, procedural efficacy, and resource utilization. The available literature demonstrates substantial heterogeneity in both pharmacological regimens and depth of sedation, and no consensus has been reached regarding the optimal anesthetic approach for AF ablation.

This review summarizes the pharmacological characteristics of the principal agents and the protocols used for deep and conscious sedation. Ultimately, the sedation strategy should be individualized according to patient characteristics, the regulatory framework, the energy source, and center-specific organizational factors.

## References

[1] Tzeis S, Gerstenfeld EP, Kalman J, Saad EB, Shamloo AS, Andrade JG (2024). 2024 European Heart Rhythm Association/Heart Rhythm Society/Asia Pacific Heart Rhythm Society/Latin American Heart Rhythm Society expert consensus statement on catheter and surgical ablation of atrial fibrillation. *Heart Rhythm*.

[2] Van Gelder IC, Rienstra M, Bunting KV, Casado-Arroyo R, Caso V, Crijns HJGM (2024). 2024 ESC Guidelines for the management of atrial fibrillation developed in collaboration with the European Association for Cardio-Thoracic Surgery (EACTS). *European Heart Journal*.

[3] Rienstra M, Tzeis S, Bunting KV, Caso V, Crijns HJGM, De Potter TJR (2024). Spotlight on the 2024 ESC/EACTS management of atrial fibrillation guidelines: 10 novel key aspects. *Europace*.

[4] Arbelo E, De Ponti R, Cohen L, Pastor L, Costa G, Hempel M (2024). Clinical and economic impact of first-line or drug-naïve catheter ablation and delayed second-line catheter ablation for atrial fibrillation using a patient-level simulation model. *Journal of Medical Economics*.

[5] Stabile G, Solimene F, Calò L, Anselmino M, Castro A, Pratola C (2014). Catheter-tissue contact force for pulmonary veins isolation: a pilot multicentre study on effect on procedure and fluoroscopy time. *Europace*.

[6] Orbán G, Boga M, Salló Z, Osztheimer I, Nagy KV, Perge P (2025). Comparison of room times between pulsed-field ablation and very high-power short-duration ablation. *Heart Rhythm O2*.

[7] Boersma LVA, Széplaki G, Dello Russo A, García-Bolao I, Efremidis M, Szegedi N (2025). Real-world experience with the pentaspline pulsed field ablation system: one-year outcomes of the FARADISE registry. *Europace*.

[8] Chun KRJ, Plank K, Neven K, Reichlin T, Blaauw Y, Hansen J (2025). Characterization of sedation strategies in real-world use of pulsed field ablation Sub-analysis of the EU-PORIA registry. *Europace*.

[9] Saglietto A, Ballatore A, Gaita F, Scaglione M, De Ponti R, De Ferrari GM (2022). Comparative efficacy and safety of different catheter ablation strategies for persistent atrial fibrillation: a network meta-analysis of randomized clinical trials. *European Heart Journal. Quality of Care & Clinical Outcomes*.

[10] Garcia R, Waldmann V, Vanduynhoven P, Nesti M, Jansen de Oliveira Figueiredo M, Narayanan K (2021). Worldwide sedation strategies for atrial fibrillation ablation: current status and evolution over the last decade. *Europace*.

[11] Palmisano P, Ziacchi M, Angeletti A, Guerra F, Forleo GB, Bertini M (2021). The Practice of Deep Sedation in Electrophysiology and Cardiac Pacing Laboratories: Results of an Italian Survey Promoted by the AIAC (Italian Association of Arrhythmology and Cardiac Pacing). *Journal of Clinical Medicine*.

[12] Chun KRJ, Miklavčič D, Vlachos K, Bordignon S, Scherr D, Jais P (2024). State-of-the-art pulsed field ablation for cardiac arrhythmias: ongoing evolution and future perspective. *Europace*.

[13] Wahedi R, Willems S, Feldhege J, Jularic M, Hartmann J, Anwar O (2024). Pulsed-field versus cryoballoon ablation for atrial fibrillation-Impact of energy source on sedation and analgesia requirement. *Journal of Cardiovascular Electrophysiology*.

[14] Schmidt B, Bordignon S, Neven K, Reichlin T, Blaauw Y, Hansen J (2023). EUropean real-world outcomes with Pulsed field ablatiOn in patients with symptomatic atRIAl fibrillation: lessons from the multi-centre EU-PORIA registry. *Europace*.

[15] Grimaldi M, Quadrini F, Caporusso N, Troisi F, Vitulano N, Delmonte V (2023). Deep sedation protocol during atrial fibrillation ablation using a novel variable-loop biphasic pulsed field ablation catheter. *Europace*.

[16] Iacopino S, Colella J, Dini D, Mantovani L, Sorrenti PF, Malacrida M (2023). Sedation strategies for pulsed-field ablation of atrial fibrillation: focus on deep sedation with intravenous ketamine in spontaneous respiration. *Europace*.

[17] Pang N, Gao J, Zhang N, Zhang B, Wang R (2022). Comparison of the Different Anesthesia Strategies for Atrial Fibrillation Catheter Ablation: A Systematic Review and Meta-Analysis. *Cardiology Research and Practice*.

[18] Araújo B, Rivera A, de Oliveira Tapioca V, Barbosa LM, Caetano L, Abreu SN (2025). Sedation vs. general anaesthesia in patients with atrial fibrillation undergoing catheter ablation: a systematic review and meta-analysis. *Europace*.

[19] American Society of Anaesthesiologists (1999). Continuum of depth of sedation: definition of general anaesthesia and levels of sedation/analgesia. https://asahq.org/standards-and-practice-parameters/statement-on-continuum-of-depth-of-sedation-definition-of-general-anesthesia-and-levels-of-sedation-analgesia.

[20] Massalha E, Dakka A, Sabbag A, Berkovitch A, Marai I, Michowitz Y (2025). Comparative analysis of anaesthesia modalities in pulmonary vein isolation: insights from a prospective multicentre registry. *Europace*.

[21] Homberg M, Betz K, van Kuijk SMJ, Luermans J, Joosten EA, Buhre W (2025). Peri-procedural respiratory complications in patients undergoing pulmonary vein isolation under procedural sedation and analgesia: Incidence and predictive factors. *International Journal of Cardiology. Heart & Vasculature*.

[22] Nordt SP, Clark RF (1997). Midazolam: a review of therapeutic uses and toxicity. *The Journal of Emergency Medicine*.

[23] Mennuni M, Bianconi L, Antonicoli S, Frongillo D, Molle G, Rossi P (2007). Fast cardiologist-administered midazolam for electrical cardioversion of atrial fibrillation. *Journal of Cardiovascular Medicine*.

[24] Poggi S, Strisciuglio T, Iuliano A, Spiniello G, Schillaci V, Arestia A (2025). Efficacy and safety of morphine during thermal catheter ablation of atrial fibrillation. *Journal of Cardiovascular Medicine*.

[25] Calvert P, Mills MT, Murray B, Kendall J, Ratnasingham J, Luther V (2025). Feasibility of pulsed field ablation for atrial fibrillation under mild conscious sedation. *Journal of Interventional Cardiac Electrophysiology*.

[26] Moravec O, Skala T, Klementova O, Skalova J, Hutyra M, Precek J (2021). General anesthesia or conscious sedation in paroxysmal atrial fibrillation catheter ablation. *Biomedical Papers of the Medical Faculty of the University Palacky, Olomouc, Czechoslovakia*.

[27] Salukhe TV, Willems S, Drewitz I, Steven D, Hoffmann BA, Heitmann K (2012). Propofol sedation administered by cardiologists without assisted ventilation for long cardiac interventions: an assessment of 1000 consecutive patients undergoing atrial fibrillation ablation. *Europace*.

[28] Sayfo S, Vakil KP, Alqaqa’a A, Flippin H, Bhakta D, Yadav AV (2012). A retrospective analysis of proceduralist-directed, nurse-administered propofol sedation for implantable cardioverter-defibrillator procedures. *Heart Rhythm*.

[29] Vevecka A, Schwab C, Forkmann M, Butz S, Issam A, Turschner O (2019). Predictive Factors and Safety of Noninvasive Mechanical Ventilation in Combination With Propofol Deep Sedation in Left Atrial Ablation Procedures. *The American Journal of Cardiology*.

[30] Foerschner L, Harfoush N, Thoma M, Spitzbauer L, Popa M, Bourier F (2022). Deep sedation with propofol in patients undergoing left atrial ablation procedures-Is it safe?. *Heart Rhythm O2*.

[31] Postema PG, Wolpert C, Amin AS, Probst V, Borggrefe M, Roden DM (2009). Drugs and Brugada syndrome patients: review of the literature, recommendations, and an up-to-date website (www.brugadadrugs.org). *Heart Rhythm*.

[32] Venn RM, Bradshaw CJ, Spencer R, Brealey D, Caudwell E, Naughton C (1999). Preliminary UK experience of dexmedetomidine, a novel agent for postoperative sedation in the intensive care unit. *Anaesthesia*.

[33] Servatius H, Küffer T, Baldinger SH, Asatryan B, Seiler J, Tanner H (2022). Dexmedetomidine versus propofol for operator-directed nurse-administered procedural sedation during catheter ablation of atrial fibrillation: A randomized controlled study. *Heart Rhythm*.

[34] Furui K, Morishima I, Kanzaki Y, Tsuboi H (2019). Coronary vasospasm caused by intravenous infusion of dexmedetomidine: Unrecognized pitfall of catheter ablation procedures of atrial fibrillation. *Journal of Cardiology Cases*.

[35] Vladinov G, Fermin L, Longini R, Ramos Y, Maratea E (2018). Choosing the anesthetic and sedative drugs for supraventricular tachycardia ablations: A focused review. *Pacing and Clinical Electrophysiology*.

[36] Oh YJ, Kim Y, Lee C, Kim DC, Doo A (2023). The effects of the administration sequence and the type of hypnotics on the development of remifentanil-induced chest wall rigidity: a randomized controlled trial. *BMC Anesthesiology*.

[37] Lameijer H, Azizi N, Ligtenberg JJM, Ter Maaten JC (2014). Ventricular Tachycardia After Naloxone Administration: a Drug Related Complication? Case Report and Literature Review. *Drug Safety - Case Reports*.

[38] Huang D, Luo Z, Song X, Zou K (2024). Global research on sufentanil use in anesthesiology from 2003 to 2023: a bibliometric analysis. *Frontiers in Pharmacology*.

[39] Jin L, Liu F, Gao H, Zheng L (2024). Efficacy and Safety of Analgesics and Sedatives during Radiofrequency Catheter Ablation of Atrial Fibrillation: A Network Meta-Analysis. *Reviews in Cardiovascular Medicine*.

[40] Bode K, Breithardt OA, Kreuzhuber M, Mende M, Sommer P, Richter S (2015). Patient discomfort following catheter ablation and rhythm device surgery. *Europace*.

[41] Pota V, Bignami E (2018). Multimodal analgesia and sedation in the electrophysiology laboratory. *Minerva Anestesiologica*.

[42] Bertolini A, Ferrari A, Ottani A, Guerzoni S, Tacchi R, Leone S (2006). Paracetamol: new vistas of an old drug. *CNS Drug Reviews*.

[43] Wick EC, Grant MC, Wu CL (2017). Postoperative Multimodal Analgesia Pain Management With Nonopioid Analgesics and Techniques: A Review. *JAMA Surgery*.

[44] Richards ND, Howell SJ, Bellamy MC, Beck J (2025). The diverse effects of ketamine, jack-of-all-trades: a narrative review. *British Journal of Anaesthesia*.

[45] Iacopino S, Filannino P, Artale P, Petretta A, Colella J, Statuto G (2024). Investigating Deep Sedation With Intravenous Ketamine in Spontaneous Respiration During Pulsed-Field Ablation. *Journal of Cardiothoracic and Vascular Anesthesia*.

[46] Elkins GR, Barabasz AF, Council JR, Spiegel D (2015). Advancing research and practice: the revised APA Division 30 definition of hypnosis. *The International Journal of Clinical and Experimental Hypnosis*.

[47] Dorta DC, Colavolpe PO, Lauria PSS, Fonseca RB, Brito VCSG, Villarreal CF (2024). Multimodal benefits of hypnosis on pain, mental health, sleep, and quality of life in patients with chronic pain related to fibromyalgia: A randomized, controlled, blindly-evaluated trial. *Explore*.

[48] Ogez D, Landry M, Caron-Trahan R, Jusseaux AE, Aubin M, Véronneau J (2024). Make me more comfortable: effects of a hypnosis session on pain perception in chronic pain patients. *Frontiers in Psychology*.

[49] Rosenbloom BN, Slepian PM, Azam MA, Aternali A, Birnie KA, Curtis K (2024). A Randomized Controlled Trial of Clinical Hypnosis as an Opioid-Sparing Adjunct Treatment for Pain Relief in Adults Undergoing Major Oncologic Surgery. *Journal of Pain Research*.

[50] Sola C, Devigne J, Bringuier S, Pico J, Coruble L, Capdevila X (2023). Hypnosis as an alternative to general anaesthesia for paediatric superficial surgery: a randomised controlled trial. *British Journal of Anaesthesia*.

[51] Scaglione M, Battaglia A, Di Donna P, Peyracchia M, Bolzan B, Mazzucchi P (2019). Hypnotic communication for periprocedural analgesia during transcatheter ablation of atrial fibrillation. *International Journal of Cardiology. Heart & Vasculature*.

[52] Nørgaard MW, Pedersen PU, Bjerrum M (2018). Understanding how patients use visualization during ablation of atrial fibrillation in reducing their experience of pain, anxiety, consumption of pain medication and procedure length: Integrating quantitative and qualitative results. *Applied Nursing Research*.

[53] Harbell MW, Dumitrascu C, Bettini L, Yu S, Thiele CM, Koyyalamudi V (2021). Anesthetic Considerations for Patients on Psychotropic Drug Therapies. *Neurology International*.

[54] Jenkins MJA, Kinsella SM, Wiles MD, Srivastava B, Griffiths C, Lewin J (2025). Peri-operative identification and management of patients with unhealthy alcohol intake. *Anaesthesia*.

[55] Gan TJ, Jin Z, Ayad S, Belani KG, Habib AS, Meyer TA (2025). Fifth Consensus Guidelines for the Management of Postoperative Nausea and Vomiting: Executive Summary. *Anesthesia and Analgesia*.

[56] Kawamura I, Miyazaki S, Nitta J, Inaba O, Takahashi A, Yamashita S (2025). Incidence of cough reflex and vagal response among various pulsed field ablation systems: a multicenter registry study. *Clinical Research in Cardiology*.

[57] Jiang R, Liu Q, Chen L, Chen S, Wang Y, Cheng H (2023). Respiratory control minimizes diaphragmatic contraction and dry cough during pulsed-field ablation of atrial fibrillation. *Europace*.

[58] Yang SS, Wang NN, Postonogova T, Yang GJ, McGillion M, Beique F (2020). Intravenous lidocaine to prevent postoperative airway complications in adults: a systematic review and meta-analysis. *British Journal of Anaesthesia*.

[59] Fredensborg MB, Fiege SB, Møller AM (2025). Anaesthetic Techniques for Cardiac Ablation-A Scoping Review Protocol. *Acta Anaesthesiologica Scandinavica*.

[60] Cho JS, Shim JK, Na S, Park I, Kwak YL (2014). Improved sedation with dexmedetomidine-remifentanil compared with midazolam-remifentanil during catheter ablation of atrial fibrillation: a randomized, controlled trial. *Europace*.

[61] Rillig A, Hirokami J, Moser F, Bordignon S, Rottner L, Shota T (2024). General anaesthesia and deep sedation for monopolar pulsed field ablation using a lattice-tip catheter combined with a novel three-dimensional mapping system. *Europace*.

[62] Poggi S, Strisciuglio T, Iuliano A, Spiniello G, Schillaci V, Arestia A (2025). Peri-procedural anesthesia and patient pain experience in pulmonary vein isolation by means of very high-power short-duration radiofrequency ablation. *Journal of Interventional Cardiac Electrophysiology*.

[63] Hinkelbein J, Lamperti M, Akeson J, Santos J, Costa J, De Robertis E (2018). European Society of Anaesthesiology and European Board of Anaesthesiology guidelines for procedural sedation and analgesia in adults. *European Journal of Anaesthesiology*.

[64] American Society of Anesthesiologists - Committee on Practice Parameters (1986). Standards for basic anesthetic monitoring. https://www.asahq.org/standards-and-practice-parameters/standards-for-basic-anesthetic-monitoring.

[65] Perel A (2011). Non-anaesthesiologists should not be allowed to administer propofol for procedural sedation: a Consensus Statement of 21 European National Societies of Anaesthesia. *European Journal of Anaesthesiology*.

[66] Occhetta E, Rillo M, Berisso MZ, Bisignani G, Forleo GB, Guerra F (2020). Quality and performance in cardiac pacing and electrophysiology. An update to the 2010 Italian Association of Arrhythmology and Cardiac Pacing (AIAC) - Italian Federation of Cardiology (IFC) Document ‘Structure and functional organization of Arrhythmology’. *Giornale Italiano Di Cardiologia (2006)*.

[67] Zylla MM, Imberti JF, Leyva F, Casado-Arroyo R, Braunschweig F, Pürerfellner H (2024). Same-day discharge vs. overnight stay following catheter ablation for atrial fibrillation: a comprehensive review and meta-analysis by the European Heart Rhythm Association Health Economics Committee. *Europace*.

